# Human embryo stem cells and DNA repair

**DOI:** 10.18632/aging.100337

**Published:** 2011-06-09

**Authors:** Marco Durante

**Affiliations:** ^1^ GSII Helmholtzzentrum für Schwerionenforschung, Planckstraβe 1, Darmstadt 64291, Germany. M.Durante@gsi.de

Pluripotent human embryonic stem cells (hESCs) are able to differentiate in all different specialized tissues [1]. Yet, as all living cells, they have to cope with endogenous or exogenous DNA damage. Their efficacy in DNA repair is somewhat controversial. Clearly hESCs can achieve the indispensable genome stability either by more effective repair or, on the contrary, by increasing apoptosis thus eliminating defective cells. Previous papers reported increased repair efficiency and involvement of the error-free homologous recombination (HR) pathway in hESCs [2, 3], and use of high-fidelity, ATM-independent non-homologous end-joining (NHEJ) [4]. Those results support the hypothesis that hESCs, which have a short G1-phase and can then use HR in most of their cell-cycle [4] are more efficient than somatic cells in DNA double-strand break (DSBs) repair.

However, Bogomazova et al. [5] now convincingly prove that hESCs use the error-prone NHEJ DNA repair pathway in G2-phase. In fact, they measured chromatid-type aberrations in hESCs and somatic human cells 2 h after exposure to x-rays. They found a similar yield of chromatid-type breaks, but a twofold increase in chromatid-type exchanges. In G2-phase, DNA DSBs can be repaired by HR or NHEJ [6], but chromatid exchanges, resulting from misrejoining of DNA breaks, are formed by NHEJ with fast kinetics [7]. Inhibition of the DNA-dependent protein kinase (DNA-PK) caused an increase in the yield of radiation-induced chromatid breaks and a decrease of chromatid exchanges in hESCs. As DNA-PK is a key factor in NHEJ [6], these results elegantly prove that NHEJ is used by hESCs in G2 to eliminate damaged cells.

The work of Bogomazova et al. [5] hence indicates that hESCs in G2 prefer a “suicide strategy” to maintain genome stability, i.e. when challenged with clastogenic agents resort to an error-prone repair mechanism, which is likely to result in cell death. Unlike previous studies pointing to alternative, high-fidelity NHEJ in hESCs [4], in these experiments [5] DSBs were introduced by ionizing radiation, a typical environmental mutagen and clastogen. These results have therefore important implications for both a mechanistic understanding of the repair mechanisms in human cells, and for the protection of embryo and fetus from exposure to ionizing radiation.
